# Robotic systems in cranial surgery: a systematic review of accuracy, clinical outcomes, and adverse events

**DOI:** 10.1007/s10143-026-04529-x

**Published:** 2026-10-10

**Authors:** Alison E. Burgess, Bailey D. Lupo, Luke P. Landry, Ben V. Peco, Nadia V. Haik, Zack Karzoun, Norris C. Talbot, James R. Bridges, Michael Folse, Ryan R. Diaz, Jamie Toms

**Affiliations:** 1 School of Medicine, Louisiana State University Health Sciences Center at Shreveport, 1501 Kings Highway, Shreveport, LA 71103 USA; 2 Department of Neurosurgery, Louisiana State University Health Sciences Center at Shreveport, 1501 Kings Highway, Shreveport, LA 71103 USA

**Keywords:** Robot, Neurosurgery, Cranial, Robot-assisted

## Abstract

Robotic-assisted surgery is becoming more prevalent in neurosurgery. Many studies have classified the advantages and disadvantages, particularly in spinal robotic surgeries. However, the use of robotics in cranial procedures has been limited to individual studies without large-scale review of the different modalities. This study is a comprehensive systematic review of the major robotics utilized in cranial neurosurgical procedures and their respective outcomes. Following Preferred Reporting Items for Systematic Reviews and Meta-Analyses (PRISMA) guidelines, a systematic review was conducted over PubMed, Cochrane, and Embase for articles published between January 2000 to February 2025. EndNote and Rayyan were utilized to organize the 1,409 identified studies and conduct a blinded multiphase process for including and excluding studies. Originally, studies were intended to be run as a meta-analysis to compare robotic modalities, but the existing data was too heterogeneous to statistically contrast outcomes. Overall, 51 studies were included, with six robots being discussed in more than one study. The use of robotics in cranial neurosurgery stretches mostly across functional neurosurgery, with different applications in brain tumor biopsies and resection, hemorrhage drainage, and endoscopic surgeries. Cranial neurosurgical procedures primarily apply robotics in the world of functional neurosurgery. Outside of this subspecialty, robotic-assisted cranial surgery remains sparse in the literature. As technology evolves, more use cases may be realized.

## Introduction

Robotic surgery is becoming more common across many fields of surgery; not only are the number of cases being performed with some amount of robotic involvement increasing, but the number of potential applications is growing as well [1]. The first “robotic” surgery was in the field of neurosurgery over three decades ago and performed a stereotactic biopsy of an intracranial lesion [2]. Today, the use of robots to stereotactically place electrodes in deep brain stimulation (DBS) and stereo-electroencephalography (SEEG) is increasingly more common. In these procedures, electrodes are placed with the help of robotic arms that guide their trajectory. Since the target locations can be deep in the brain and as small as a few millimeters, correct placement without proper navigation and guidance can prove difficult. The robots utilize navigation systems that take advantage of preoperative neuroimaging to create the safest and most effective insertion trajectories. This guidance allows for a highly accurate, patient-specific approach which maximizes effectiveness and reduces the risk of complications [3]. Because robotic trajectory planning depends heavily on preoperative imaging and registration accuracy, anatomical landmarks that remain recognizable intraoperatively, including superficial cortical veins, may help compensate for brain shift and improve spatial orientation during cranial procedures [4, 5]. Other cranial procedures that commonly use robotic assistance include laser interstitial thermal therapy (LITT) and responsive neurostimulation (RNS) electrode placement, as well as procedures such as biopsy of brain tumors in difficult-to-reach locations.

Given these robots’ potential to improve patient outcomes, they have become increasingly valuable in the operating room. Robotic applications in neurosurgery are only in their infancy, and future directions focus on increasing the level of automation to simplify and shorten these procedures. Robotic systems in neurosurgery have several potential advantages, including improved accuracy, precision, and reproducibility. However, these systems have several potential limitations including high acquisition and maintenance costs, significant learning curves, and the need for specialized training. While current applications are largely centered around stereotaxis, further advancement could allow other areas of neurosurgery to be improved as well.

There is no shortage of data outlining the effectiveness of robotics in neurosurgery, particularly in spinal surgery, but there is a lack of literature that gives a clear overview of the current state of cranial robotic surgery. This paper is a systematic review to comprehensively assess all current applications of robotics within cranial neurosurgical procedures. This study aims to discuss what metrics cranial robots are improving in the field of neurosurgery and identify sources with the most impactful data to estimate what the future of cranial robotic surgery could look like.

## Methods

### Eligibility criteria

To collect an adequate sample size, inclusion and exclusion criteria were kept broad to maximize the number of studies identified. The inclusion criteria included (1) any study involving cranial robotic surgery published between January 2000 and February 2025, (2) surgeries for any diagnosis across all age groups, and (3) a minimum of 3 patients and results of surgery were recorded. Exclusions were made at different stages in the systematic review and included (1) any duplicate study was only counted once, (2) any non-original data, (3) any study not including intracranial robotic surgery, (4) any animal or cadaveric study, (5) studies where full access was not obtainable, and (6) studies where the full-text version could not be found in plain English.

### Literature search instructions

**Fig. 1:**
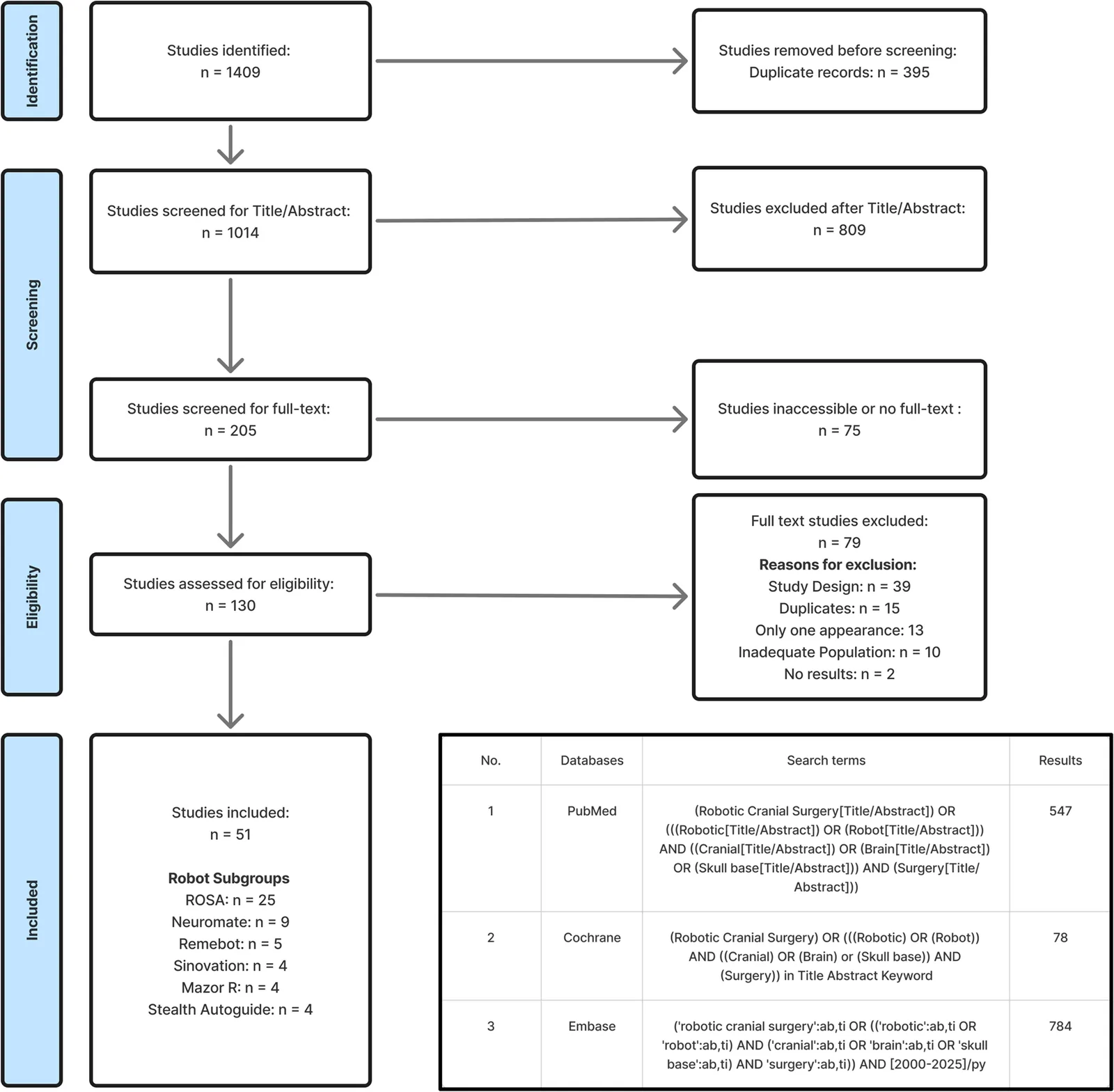
PRISMA flow chart depicting search criteria and steps of the systematic review process

A wide range of search criteria was used to obtain as many studies as possible. This search criteria used logic statements which are defined in Fig. 1 to include any study that could possibly involve robotic cranial surgery. The following databases were queried using the search criteria: Pubmed, Cochrane, and Embase. Filters were used in each database to obtain only studies published from January 2000 to February 2025. The search was limited to its broadest subset and was conducted following the Preferred Reporting Items for Systematic Reviews and Meta-Analyses (PRISMA) guidelines [6].

### Study selection and data extraction

Identified studies were downloaded and imported into EndNote and de-duplicated. Once duplicates were removed, Rayyan was used for the screening process, allowing reviewers to be blinded. The first stage of screening included 9 independent reviewers randomly reviewing abstracts, with each study requiring at least two votes. If reviewers disagreed, a third reviewer blindly reviewed the abstract and made a final decision. The second screening phase was full-text screening of all accepted studies from phase 1 and was carried out by 8 independent reviewers in groups of 3 to establish a majority vote. Throughout all phases, studies were assessed and voted on based upon the previously established inclusion and exclusion criterion. The studies accepted from phase 2 were then analyzed, and data was input into Excel spreadsheets.

## Results

### Study characteristic

A total of 1,409 studies were identified during the database search conducted, including articles from January 2000 to February 2025. Duplicate entries were identified and excluded (*n* = 395). The remaining studies (*n* = 1,014) proceeded to the screening phase, where titles and abstracts were first reviewed, followed by full-text assessment. During the title and abstract screening, 809 studies were excluded based on the predefined criteria. Subsequently, 205 studies were screened for full-text accessibility, and 75 were found to be inaccessible or lacking a full-text version. The remaining 130 studies were thoroughly assessed for eligibility, resulting in the exclusion of 79 studies for various reasons, including study design (*n* = 39), remaining duplicates (*n* = 15), the robot only appearing once in the literature (*n* = 13), inadequate population size (*n* = 10), and absence of results (*n* = 2). A total of 51 studies were ultimately included in this systematic review, encompassing a wide range of cranial robotic systems. These included ROSA (Zimmer Biomet, Warsaw, IN, USA) (*n* = 25), Neuromate (Renishaw plc, Gloucestershire, UK) (*n* = 9), Remebot (Beijing Baihui Weikang Technology Co., Ltd., Beijing, China) (*n* = 5), Sinovation (Sinovation Medical Technology Co., Ltd., Beijing, China) (*n* = 4), Mazor Renaissance (Mazor Robotics Ltd., Caesarea, Israel) (*n* = 4), and Stealth Autoguide (Medtronic plc, Dublin, Ireland) (*n* = 4). 13 robotic platforms only appeared in a single publication and were subsequently excluded from tabulation to maintain focus on systems with sufficient representation in the literature to allow meaningful comparison. CyberKnife studies were excluded due to radiosurgical workflows and outcomes differing significantly when compared to neurosurgical robotics. The study selection workflow and search criteria is highlighted in Fig. 1.

### Robotic devices

#### Mazor Renaissance

The Mazor Renaissance robot was evaluated in four studies, three of which examined DBS lead placement (Table 1). Accuracy was consistently comparable to non-robotic approaches, though operative time findings were mixed, with two studies reporting shorter procedure times and one reporting longer times. One study also noted a lower infection rate in the robotic cohort. A fourth study assessed the robot for brain mass biopsy, finding it combined the stability of a bone-mounted frame with the flexibility of a frameless system, with one epidural hematoma complication [7–10]. Overall, the Mazor Renaissance has shown efficacy in DBS as an alternative for accurate lead placement, with some evidence of reduced operative time.

**Table 1:** Mazor Renaissance Studies

Author	Procedure	Number of Patients	Age	Outcome	Results/Conclusion
Fenoy and Conner (2022) [7]	DBS Lead Placement	34	65	Operating Time and Accuracy	This study compared the Mazor Renaissance to non-robotic approaches. It showed that accuracy was comparable, but found differences in operation time, indicating that the framed, non-robotic approach was significantly faster.
Grimm et al. (2015) [10]	Biopsy	37	57.4	Efficiency and Safety	The study showed that the robot’s utilization in biopsying supratentorial lesions provided stability comparable to bone-mounted setups while also adding the flexibility and maneuverability of a frameless system. One epidural hematoma complication.
Ho et al. (2019) [8]	DBS	20	67.4	Operative Time, Accuracy, Complications	Accuracy was not assessed to a suitable degree. There was a notable decrease in operative time indexes in the robotic cohort. In the nonrobotic cohort, there were three complications noted; for the robotic cohort one complication was reported.
Vansickle et al., (2019) [9]	DBS	128	64.6	Radial Error, Operative Time, Adverse Events	Mazor Renaissance operative time was shown to be on the lower end of non-robotic asleep cases and significantly lower than awake DBS lead placements. Accuracy was not sacrificed in robotic cases and infections were found to be lower in the robotic group.

*DBS* Deep Brain Stimulation

#### Sinovation

The Sinovation robot was evaluated in four studies (Table 2). Two assessing DBS lead placement, one biopsy, and one hematoma drainage. Both DBS studies found accurate, safe lead placement, with clinical seizure reduction reported in one [11, 12]. In biopsy, the robot significantly reduced target and entry-point error alongside a lower complication rate [13]. For brainstem hematoma drainage, the robot outperformed conservative management, though this comparison was made against a non-surgical rather than a non-robotic surgical group [14].

**Table 2:** Sinovation Robot Studies

Author	Procedure	Number of Patients	Age	Outcome	Results/Conclusion
Hu et al. (2022) [13]	Biopsy	104	45.3	Accuracy, Operative Time, Complications	The robot group had a significant decrease in target and entry point error and operative time. It was also found to have a lower rate of complications.
Mei et al. (2022) [11]	DBS	35	61.7	Accuracy and Precision	The authors found that awake frameless robot-assisted DBS surgery was comparable to conventional frame-based technique in the accuracy and precision of lead implantations.
Tang et al. (2024) [14]	Brainstem Hematoma Drainage	51	55	Complications and GCS, NIHSS, and mRS scores after 6 months	Use of the Sinovation robot for brainstem hematoma drainage was shown to decrease mortality rates and improve prognosis (using the NIHSS, GCS, and mRS scales) compared to medically managed group.
Wang et al. (2023) [12]	DBS of CMT	10	22	Accuracy and Clinical Results	Placement of DBS leads in the CMT was both precise and safe. The procedure was also effective in reducing seizures.

*DBS* Deep Brain Stimulation, *GCS* Glasgow Coma Scale, *NIHSS* National Institutes of Health Stroke Scale, *mRS* Modified Rankin Scale, *CMT* Centromedian Thalamic Nucleus

#### ROSA

ROSA-assisted DBS was evaluated in nine studies, focusing on accuracy, safety, procedure time, and complications. Most studies found accuracy comparable or superior to frame-based methods [15–20], and robotic DBS was considered as safe as frame-based surgery [19, 21]. Procedure time findings were mixed, with some studies reporting improved efficiency and others reporting longer operative times and higher complication rates [19, 20]. Asleep ROSA DBS was faster than awake ROSA DBS without any difference in accuracy or outcomes [15, 22]. Complication rates were low, with infection the most commonly reported adverse event, and postoperative UPDRS scores were comparable to conventional registration techniques [21, 23]. Overall, ROSA-assisted DBS appears equally or more accurate and safe than traditional surgery, though its effect on operative time remains unclear.

Seven studies evaluated ROSA for SEEG electrode placement, examining accuracy, procedure time, complications, and seizure outcomes. Accuracy was comparable to or better than frame-based surgery, and procedure time was consistently reduced [24–29]. Complication rates were low overall, with one study reporting a 4% rate (two subdural and two intraparenchymal hematomas) [27]. The one study assessing seizure outcomes reported favorable seizure control [28]. A separate study of combined DBS/SEEG implantation concluded that further research is needed [30]. Overall, ROSA-assisted SEEG appears accurate, with decreased operative time and low complication rates.

Four studies assessed ROSA for cranial biopsy, focusing on diagnostic rate, complications, and operative time. Operative time results were mixed with one study reporting shorter times than frameless biopsy, while another reported longer times. but no differences in complication rates were noted compared to frameless methods, and one study reported a higher diagnostic rate with ROSA [31, 32]. All four studies concluded ROSA is safe and effective for cranial biopsy [18, 31–33].

Regarding the less common procedures mentioned in Table 3, using ROSA had minimal complications, reduced operation time, high accuracy, and overall the performance was comparable to the frame-based methods [30, 34–39].

**Table 3:** ROSA Robot Studies

Author	Procedure	Number of Patients	Age	Outcome	Results/Conclusion
Doddamani et al. (2021) [34]	VPS	3	1, 32, 38	Success	Robotic assisted ventricular shunt placement was shown to be safe, effective, and accurate, even in the presence of slit-like ventricles.
Faraji et al. (2020) [17]	DBS	20	58.55	Accuracy, Total Case Time	DBS placement with ROSA has the potential to enhance accuracy and reduce operator error associated with traditional frame-based surgery.
Feng et al. (2023) [18]	Biopsy	407	42.1	Diagnostic Rate, Post-Op Complication Rate, Mean Duration	Comparative study of ROSA, CAS-R-2, and Remebot systems. Diagnostic rate, safety, and efficacy of robot-guided biopsies depend on lesion location and size, surgeon experience, and number of biopsies performed at the center. The Remebot significantly reduced procedure time compared to the other two robotic systems: CAS-R-2 and ROSA. There were no significant differences in diagnostic rates between the robotic systems and all demonstrated high safety and effectiveness.
Goia et al. (2019) [19]	DBS	24	61	Accuracy, Duration of Procedure, Complications	Rosa demonstrated equal or better accuracy, efficiency, and achieved safe electrode placement compared to frame based surgery.
González-Martínez et al. (2016) [27]	SEEG	100	33.2	Accuracy, Complications, Time of Procedure	Complication rate of 4%, which included 2 subdural hematomas and 2 intraparenchymal hematomas. Robot-assisted implantations reduced procedure time significantly compared to frame-based. The robotic system proved to be safe, accurate, and efficient in epilepsy surgery.
Gupta et al. (2021) [31]	Biopsy	22	9.1	Post-Operative Outcomes, Diagnostic Yield, Operative Time	Pediatric brainstem and thalamic lesions can be biopsied safely with shorter operative times using the ROSA robotic system.
Jin et al. (2020) [15]	DBS	153 total 58 General Anesthesia (GA) 95 Local Anesthesia (LA)	63.5 + 6.6 General Anesthesia 63.2 + 9.7 Local Anesthesia	Accuracy, UPDRS & UPDRS-III, Simulation parameters, Complications, Operative metrics, Clinical Outcomes	The GA group and LA group did not differ in accuracy or intraoperative electrophysiological signal length. The investigators designed the Tao’s DBS surgery scale to evaluate the quality of DBS surgery. With GA, the procedure time was significantly reduced and the Tao’s DBS surgery scale was higher when compared with LA.
Karas et al. (2020) [35]	RNS (Responsive Neurostimulation)	10	25–63	Accuracy, Seizure Outcomes	ROSA demonstrated high accuracy, especially when the patient was in a prone position compared to a semi-sitting position. Seizure outcomes were consistent with traditional methods. No complications were reported in study.
Lefranc et al. (2017) [22]	DBS	23	60	UPDRS-III & IV, Adverse Events, Cognitive & Neuropsychiatric Effects, Therapeutic Window	Robot-assisted STN-DBS surgery under GA and LA had similar outcomes regarding STN identification, lead positioning, and motor results.
Liang et al. (2022) [36]	ICH Drainage	71	57.24	Complications, Operative Time, Clinical Indexes	When evacuating an intracerebral hematoma, ROSA reduced the procedure and postoperative extubation time, as well as lowered the risk of infection, rehemorrhage, and inflammatory level.
Liu et al. (2020) [16]	DBS	144 total 91 X-Ray 53 O-Arm	53 ± 13 X-Ray 55 ± 12 O-Arm	Accuracy	In ROSA-assisted DBS surgery there was no significant difference in accuracy confirmation with intraoperative X-ray vs. O-arm imaging compared to postoperative CT scans. The ROSA system achieved submillimeter placement accuracy.
Luh et al. (2025) [37]	ICH Aspiration	7	18+	Operation Time, Functional Outcomes, Complications	ROSA-assisted ICH aspiration was found to be safe and effective for reducing hematoma volume and associated with favorable functional outcomes. No complications were observed.
Mallereau et al. (2022) [32]	Biopsy	314	59.61	Complications, Diagnostic Rate, Operation Time	Robotic-assisted biopsies yielded a higher diagnostic rate than frameless biopsies. No significant difference in complication rate. The procedure time using ROSA was longer than frameless biopsy.
McGovern et al. (2019) [28]	SEEG	57	12	Complications, Seizure Outcomes, Operation Time	Using ROSA for SEEG reduced operative time, demonstrated a low complication rate, and had good overall seizure outcomes.
Nelson et al. (2020) [24]	SEEG	19	13	Operative Time, Complications, Surgical Outcomes	No complications were observed intraoperatively or perioperatively when using ROSA for SEEG. Using the robot reduces the operation time and provides high accuracy and safety.
Ollivier et al. (2017) [25]	SEEG	66	28	Complications, Accuracy, Operation Time	ROSA-assisted SEEG is safe and accuracy is comparable to frame-based surgery. The operation time is reduced with an overall reported complication rate of 3%.
Paff et al. (2019) [20]	DBS	20	63.5	UPDRS, Operative times, Complication rates, Accuracy	Robot-assisted DBS surgery demonstrated comparable improvements in motor scores, reductions in mean LEDD, and accuracy. However, operative times were longer and there was a slightly higher complication rate.
Rubino et al. (2021) [38]	Laser Interstitial Thermal Therapy (LITT) & Stereotactic Biopsies	60 total 27 biopsies 43 MRI-guided LITT	60.3	Diagnostic yield, Intraoperative & Postoperative Complications	Diagnostic yield of 98.5% was reported. No permanent morbidity or intraoperative complications were reported. As for postoperative complications, there were two deaths. The study concluded robot-assisted LITT and biopsy is safe and accurate.
Sheth et al. (2023) [30]	SEEG and DBS combined implantation surgery	3	N/A	Accuracy, Intraoperative Complications,	Only one patient experienced intraoperative complications. There were challenges when combining SEEG and DBS techniques and the method should be further investigated.
Spyrantis et al. (2018) [29]	SEEG	5	31	Accuracy, Complications, Operative time	Robot-assisted implantation had higher accuracy than other methods. No complications were observed and this procedure was considered safe.
Spyrantis et al. (2019) [26]	SEEG	19	N/A	Accuracy, Complications	ROSA-assisted SEEG implantation using CT-frame referencing and CT-laser based referencing demonstrated higher accuracy than 3.0-T MRI-laser based referencing. No major complications or new neurological deficits were reported.
Terrier et al. (2018) [33]	Biopsy	60	59.6	Complications, Diagnosis Yield, Operation Time	All biopsies performed were diagnostic. 10% of patients had asymptomatic bleeding post-op and permanent clinical worsening was reported in 3%. No reported infections or mortality. Overall the procedure was deemed safe and efficient.
Wang et al. (2022) [39]	Simulation assisted, Robot-assisted Radiofrequency Ablation (S-RARFA)	3	28yo, 45yo, 9yo	Complications, Accuracy	No postoperative complications or new deficits were observed. S-RARFA was concluded to be very precise, cause minimal damage, and achieve good seizure outcomes.
Wu et al. (2024) [21]	DBS	40	78.4	Postoperative Side Effects, Postoperative Clinical Assessment, Survival Status, Operation time	Study included 22 ROSA-assisted and 7 Remebot-assisted DBS procedures. One patient developed an infection, but no other postoperative complications were observed. Based on the results of this study, robot-assisted DBS is safe and effective for elderly patients with Parkinson’s disease.
Xu et al. (2018) [23]	DBS	71 Total Group I: 33 conventional registration Group II: 38 modified registration methods	Group I: 64.7 Group II: 65.0	Accuracy, UPDRS, UPDRS-III	The modified registration method studied could reduce registration errors and electrode vector errors. The UPDRS or UPDRS-III score at 10 weeks postoperative was similar to conventional registration. The long-term effects of the method need to be further studied.

*VPS* ventriculoperitoneal shunt, *DBS* deep brain stimulation, *SEEG* stereoelectroencephalography, *GA* General Anesthesia, *LA* Local Anesthesia, *RNS* Responsive Neurostimulation, *LITT* Laser Interstitial Thermal Therapy, *S-RARFA* Simulation Assisted, Robot-assisted Radiofrequency Ablation, *UPDRS* Unified Parkinson’s Disease Rating Scale, *STN* Subthalamic Nucleus, *ICH* Intracerebral Hemorrhage, *LEDD* Levodopa Equivalent Daily Doses

#### Remebot

Remebot was evaluated across biopsy, hematoma aspiration, and Ommaya reservoir implantation studies (Table 4). A novel technique, robot-assisted visualized minimally invasive aspiration (RAVMIA), improved clinical outcomes for intracerebral hemorrhage [40]. Two biopsy studies found the robot reliably reached target lesions with complication rates comparable to conventional methods [41, 42]. One study discussed potential technological upgrades to further improve safety and efficiency, and the robot was also shown to be highly accurate and minimally invasive for Ommaya reservoir implantation [43, 44].

**Table 4:** Remebot Robot Studies

Author	Procedure	Number of Patients	Age	Outcome	Results/Conclusion
Luo et al. (2024) [40]	ICH	3	56	Effectiveness at treating ICH	The Remebot^®^ RM-100was shown to significantly increase intraoperative hematoma evacuation rate, decrease the EOT residual ICH volume, and shorten hospital stay, while not prolonging surgery time.
Ma et al. (2022) [43]	DBS	30	56.7	Accuracy Compared to Novel Frameless Device	This study showed no significant difference between conventional DBS procedures and Remebot DBS with assistance from a novel frameless videometric registration system.
Liu et al. (2021) [44]	Ommaya Reservoir implant	100	18.6	Implant Characteristics Compared to Conventional Methods of Implantation	This study analyzed metrics associated with the Ommaya reservoir implantation procedure and reported smaller burr hole diameter, shorter operation time, less intraoperative blood loss, fewer complications, and a perfect rate of successful punctures, with all 50 reaching the ventricle on the first attempt.
Liu et al. (2021) [41]	Biopsy	700	47.6	Operation time, Accuracy, Complications	All metrics were acceptable. The study showed that robotic biopsy was an acceptable approach for brain biopsies.
Li et al. (2023) [42]	Biopsy	33	22.5	Diagnostic Yield and Complications	The Remebot robotic system was as effective and as safe as the traditional frame-based stereotactic procedures for brainstem tumor biopsies.

*ICH* Intracerebral Hemorrhage, *EOT* End of Treatment, *DBS* Deep Brain Stimulation

#### Stealth autoguide

Four studies evaluated the Medtronic Stealth Autoguide (SA), covering stereotactic biopsy, fence-post tube placement, and multi-procedure use (Table 5). In biopsy, the SA showed complication rates similar to traditional stereotactic methods; one study noted a lower diagnostic yield, attributed to a lower threshold for pursuing robotic-assisted biopsy rather than a device limitation [45, 46]. The SA’s precision also enabled fence-post tube placement for tumor margin demarcation with accuracy not achievable manually [47]. A 2024 study evaluating the SA across SEEG, biopsy, and LITT found consistently strong effectiveness, accuracy, and reliability regardless of procedure [48].

**Table 5:** Stealth Autoguide Robot Studies

Author	Procedure	Patients	Age	Outcome	Results/Conclusion
Atai and Mehta (2024) [48]	SEEG, Biopsy LITT	75	Median: 45 (22–68)	Accuracy and success of procedure	The authors found that the SA cranial robotic system was highly efficient and accurate across the main indications of SEEG, LITT, and stereotactic biopsy.
Koizumi et al. (2021) [47]	Fence-Post Tube Placement	3	42	Success of novel application	3 patients underwent this procedure and no patient experienced adverse events or any complications.
Ljungqvist et al. (2024) [45]	Biopsy	78	62	Diagnostic Yield and Complications	Diagnostic yield was lower in the group that used the stealth autoguide; Rates of complications were similar.
Rotim et al. (2021) [46]	Biopsy	3	60.3	Safety and Effectiveness	All three tumors were successfully identified; this study indicated that precision may be improved without a concurrent increase in operative time.

*SEEG* Stereoelectroencephalography, *LITT* Laser Interstitial Thermal Therapy, *SA* Stealth Autoguide

#### Neuromate

Nine studies evaluated the Renishaw Neuromate robot, primarily for DBS lead placement, with fewer studies on biopsy and LITT (Table 6). Across DBS studies, targeting accuracy was consistently high, with mean errors ranging from approximately 0.6–1.7 mm depending on cohort and registration technique, and several studies reported accuracy comparable to standard frame-based methods [49, 51–53, 55]. Frameless Neurolocate registration achieved accuracy similar to traditional frame-based approaches in both pediatric and adult cohorts [54, 55]. Clinical outcomes following DBS were favorable on the Burke-Fahn-Marsden Dystonia Rating Scale and UPDRS III [51, 56]. Major complications including hemorrhage, stroke, and infection occurred at rates comparable to traditional neurosurgery. Biopsy studies reported diagnostic yields of 18/22 and 10/11 patients with no surgical complications [49, 50]. One study of MRI-guided LITT for hypothalamic hamartomas reported favorable outcomes with acceptable targeting error and most patients achieving seizure freedom, despite some complications [57].

**Table 6:** Neuromate Robot Studies

Author	Procedure	Number of Patients	Age	Outcome	Results/Conclusion
Varma and Eldridge (2006) [49]	DBS Lead Placement, Brain Lesion Biopsy	153	N/A	Accuracy, Clinical Outcomes	The Neuromate Robot System demonstrated a high degree of accuracy. 111 patients had DBS lead placement targeting STN and showed a mean error of 1.7 mm. Clinically, the Parkinson’s disease patients showed significant improvement in tremors, rigidity, bradykinesia, gait, and postural instability. 22 patients underwent stereotactic surgery for mass biopsy, with 18 yielding a diagnosis and a mean registration error of 1.6 mm.
Dawes et al. (2018) [50]	Brain Tumor Biopsy	11	2–15 yo	Accuracy, Clinical Outcomes, Complications	The Neuromate Robot System successfully obtained brainstem tumor biopsies in the pediatric population. 10 of 11 biopsies in the study yielded a diagnosis from the pons and midbrain. Target point localization error was calculated with a median error of 2.7 mm. There were no surgical complications noted.
Candela et al. (2018) [51]	DBS Lead Placement	6	7–16 yo	Accuracy, Clinical Outcomes, Complications	Six pediatric patients diagnosed with dystonia underwent DBS lead placement with the average error from the target point being 1.24 mm. They showed clinical improvement in 6-month follow-up with significant improvement in motor function, myoclonus, the Burke-Fahn-Marsden Dystonia Rating Scale, and the Unified Motor-Rating Scale. One patient experienced a medial tip deviation of 4 mm that was not clinically significant.
Ribault et al. (2020) [52]	DBS Lead Placement	20	N/A	Accuracy, Clinical Outcomes	No significant difference was found between robot and traditional DBS lead placement. Operating time was longer with robot-assisted surgeries. The robot-assisted surgeries and traditional surgeries both had similar rates of complications.
Moran et al. (2020) [53]	DBS Lead Placement	113	Mean Age 64 +/- 9 yo	Accuracy	In an “asleep” cohort of 226 electrodes, the mean radial error was 0.6 ± 0.33 mm. In comparison, their mean 3D vector error was 0.78 ± 0.37 mm. No significant difference in the accuracy measurement was noted between left- and right-sided electrode placement.
Moran et al. (2021) [56]	DBS Lead Placement	152	Mean Age 60 +/- 9 yo	Clinical Outcomes	Reviewed results from bilateral STN electrode implantation for the treatment of PD using “asleep” DBS with the Neuromate robot at 1 year and showed improvements on UPDRS III assessments.
Candela-Canto et al. (2022) [57]	LITT	11	Mean Age 6.4 yo; 15 months − 17 yo	Accuracy, Procedural Duration, Clinical Outcomes, Complications	The Neuromate Robot System demonstrated sufficient accuracy and positive clinical outcomes in MRgLITT procedures. There was a target point error of 1.69 ± 0.98 mm. The mean time duration for the procedure was 6.8 ± 1.4 h. Six patients were seizure-free following the first laser ablation and four patients were seizure free after the second ablation. Complications included contralateral hemiparesis (1), transient oculomotor palsy (2), and one patient who had symptoms of hyperphagia, somnolence, and SIADH.
David et al. (2024) [54]	DBS Lead Placement	24	Mean Age 11 yo; 4–18 yo	Accuracy	The Neuromate Robot System demonstrated safety and accuracy comparable to standard frame-based techniques in the pediatric population for DBS lead placement. The x-, y-, and z-axis error rates for the Neuromate System with Neurolocate were 0.35, 0.75, and 0.9, compared to 0.30, 0.95, and 1.1 using standard frame-based techniques.
Fontaine et al. (2023) [55]	DBS Lead Placement	17	N/A	Accuracy	The Neuromate Robot System demonstrated a relatively high degree of accuracy for DBS lead placement of 0.7 mm +/- (0.4 mm), with the advantage of being less invasive and uncomfortable than other traditionally used methods.

*DBS* Deep Brain Stimulation, *STN* Subthalamic Nucleus, *TPLE* target point localization error, *PD* Parkinson’s Disease, *UPDRS* Unified Parkinson’s Disease Rating Scale, *MRgLITT* MRI-guided laser interstitial thermal therapy

## Discussion

This study is a comprehensive systematic review of the use of various robots in cranial procedures. Major outcomes that were reported included accuracy, operative time, and complications. Widely employed in neurosurgical procedures, ROSA was the most studied robot with clear utilization in functional neurosurgery. Overall, the ROSA, Neuromate, and Remebot systems emerged as the most extensively studied platforms, accounting for the majority of literature. Many of the other robots followed suit performing the same procedures with occasional applications in hematoma drainages, tumor biopsies, and tumor excision. Ultimately, these cranial robots are used to provide a stable and accurate arm to target a specific point.

The robotic systems discussed showed the greatest advantage in procedures with multiple stereotactic trajectories, specifically DBS and SEEG. In these procedures the robotic systems provided consistent high accuracy with lower complication rates and often reduced operating times. As for biopsy procedures, the robotic assistance was shown to be safe and effective, with high diagnostic yields and improved precision, especially in difficult to access lesions. It was shown that there are also promising outcomes in other emerging applications, such as hematoma evacuation, ventricular catheter placement, and LITT, but these procedures are still supported by limited evidence. Overall, the literature reviewed suggests that cranial robotic systems provide the greatest benefit in procedures where stereotactic accuracy and trajectory planning are critical to surgical success.

The potential benefits these robots offer are most appreciated in cases where multiple targeting points are required. Setting up the robot takes time that otherwise could be spent doing the procedure. As a result, the dominant use case of these robots is in cases that require multiple lead placements due to the advantage of having a system that establishes trajectories between the entry site and the target automatically and accurately. Cranial robots can quickly move between each point, saving operative time and sometimes removing the need for frame-based strategies. In these procedures, robotic systems have proven to be both accurate and efficient. Conversely, if only one point is targeted, the cost of using robotic assistance may not outweigh the benefits.

In terms of usage across subspecialties, robotic assistance is most commonly used in functional neurosurgery, providing accuracy and increased efficiency involved in lead placements. However, robotics have yet to be established in standard treatment plans within other neurosurgical subspecialties. While robots are regularly utilized in lead placement, there are other applications in development which suggest that neurosurgeons continue to search for new ways to appreciate the advantages of robot assisted surgery. In tumor biopsies, hematoma drainages, and ventricular catheter placement, cranial robotics are mainly used to provide a stable, accurate arm to target objects. Otherwise, cranial robotics have yet to find a foothold outside of functional neurosurgery.

Beyond the accuracy and efficiency of which cranial robots have proven successful, several practical factors will determine whether these systems are able to achieve broader adoption. High costs of these robotic platforms remain a significant barrier, especially for centers with lower case volumes where the expense may take longer to be offset. This burden is also compounded by a well-known learning curve with any robotic procedures including cranial neurosurgical procedures [58]. Both of these factors are heavily intertwined with the workflow integration of the robotic systems. Preoperative planning, set up time, training staff, and more add to requirements needed to implement these robotic systems across neurosurgical specialties as a whole and may not justify the acquisition and maintenance for centers with smaller case volumes. As previously mentioned, registration accuracy and brain shift compound upon this burden. Intraoperative CT or MRIs may be used in these robotic cases to maintain the high level of accuracy a robotic system provides, but these strategies also add to the economic burden as well as the time cost intraoperatively. Overall, these factors taken into account with the findings of this study suggest that standardized reporting metrics are needed in the cranial neurosurgical robotic space that not only address accuracy and precision but also cost, learning curve length, and operative efficiency.

Based on the findings of this study and the most common applications, new robots and applications are necessary to allow for more comprehensive incorporation of these systems in cranial neurosurgery. The development of remote-based robotics for spinal surgery suggests that these technologies may translate to cranial procedures in the future [59]. Furthermore, advancements in technology could allow for greater utility of robots in minimally invasive procedures. The Da Vinci system commonly employed in other surgical fields suggests the possible benefits of a robotically-controlled system to remove the microscope and instead operate with fine movements at a console but has yet to be implemented into neurosurgery [60]. In neuro-oncology, future robotic platforms may also intersect with emerging precision-treatment paradigms, including targeted delivery strategies and nanomedicine-based approaches for glioblastoma, although such applications remain speculative and are not yet supported by clinical robotic-surgery data [61–63]. While cranial robotics are advantageous for functional, multi-targeted procedures, their associated limitations indicate that further innovation is required to improve patient outcomes across the specialty.

## Conclusion

This systematic review demonstrates that robotic technology has established a significant presence in functional neurosurgery, with evidence supporting its use in DBS, SEEG, and stereotactic biopsy procedures. Across 51 studies examining six distinct robotic systems, the data shows that cranial surgical robots achieve accuracy comparable to or exceeding traditional frame-based methods, with several studies reporting reduced operative times and lower complication rates. As robotic technology continues to evolve, future research should focus on defining its role beyond established stereotactic applications.

### Limitations

Several limitations must be acknowledged in this systematic review. The heterogeneity of the included studies precluded meta-analysis, with significant variation in outcome measures, patient populations, and procedural techniques across different robotic systems. Many studies, particularly those examining individual robotic systems, had small sample sizes and lacked randomized controlled designs, limiting the generalizability of findings. The absence of standardized reporting metrics for accuracy, operative time, and complications made direct comparisons between robotic platforms challenging. Additionally, the exclusion of robotic platforms represented by only a single publication may have introduced selection bias and limited representation of emerging technologies with a smaller existing evidence base. Furthermore, 75 studies identified during the search were excluded due to inaccessibility or lack of full-text availability, potentially introducing publication bias. The review was limited to English-language publications, which may have excluded relevant studies from international databases. Several studies did not specify the exact robotic system used or provided insufficient detail regarding surgical technique, and follow-up periods varied considerably across studies, making it difficult to assess long-term outcomes consistently. Finally, the rapid evolution of robotic technology means that newer systems and updated software versions may have improved capabilities not reflected in the older literature included in this review.

## Data Availability

No datasets were generated or analysed during the current study.
